# Soft Tissue Vibrations in Running: A Narrative Review

**DOI:** 10.1186/s40798-022-00524-w

**Published:** 2022-10-22

**Authors:** Marie-Caroline Play, Robin Trama, Guillaume Y. Millet, Christophe Hautier, Marlène Giandolini, Jérémy Rossi

**Affiliations:** 1 Univ Lyon, UJM-Saint-Etienne, Inter-University Laboratory of Human Movement Biology, EA 7424, 42023 Saint-Étienne, France; 2 Univ Lyon, University Claude Bernard Lyon I, Inter-University Laboratory of Human Movement Biology, EA 7424, 69622 Lyon, France; 3 Institut Universitaire de France (IUF), Paris, France; 4 Amer Sports Footwear Innovation and Sport Sciences Lab, Salomon SAS, Annecy, France; 5 LIBM, Campus Santé Innovations, 10 chemin de la Marandière, 42270 Saint-Priest-en-Jarez, France

**Keywords:** Accelerometer, Compression, Fatigue, Footwear, Soft tissue vibrations

## Abstract

During running, the human body is subjected to impacts generating repetitive soft tissue vibrations (STV). They have been frequently discussed to be harmful for the musculoskeletal system and may alter running gait. The aims of this narrative review were to: (1) provide a comprehensive overview of the literature on STV during running, especially why and how STV occurs; (2) present the various approaches and output parameters used for quantifying STV with their strengths and limitations; (3) summarise the factors that affect STV. A wide set of parameters are employed in the literature to characterise STV. Amplitude of STV used to quantify the mechanical stress should be completed by time–frequency approaches to better characterise neuromuscular adaptations. Regarding sports gear, compression apparels seem to be effective in reducing STV. In contrast, the effects of footwear are heterogeneous and responses to footwear interventions are highly individual. The creation of functional groups has recently been suggested as a promising way to better adapt the characteristics of the shoes to the runners’ anthropometrics. Finally, fatigue was found to increase vibration amplitude but should be investigated for prolonged running exercises and completed by an evaluation of neuromuscular fatigue. Future research needs to examine the individual responses, particularly in fatigued conditions, in order to better characterise neuromuscular adaptations to STV.

## Key Points

- To provide a good overview of soft tissue vibrations, triaxial accelerometers should be used, so that all components can be accurately measured and analysed.
- Compression apparels generally decrease soft tissue vibrations whereas the effects of footwear are highly subject-specific.
- Fatigue increases soft tissue vibrations but when identified, an evaluation of neuromuscular fatigue should be undertaken.

## Introduction

The principal aim of Research & Development departments in sport companies is to guarantee comfort, limit the rate of muscle injury and improve performance through the development of innovative products for active population as well as athletes. Accordingly, impact-related stress applied to the musculoskeletal system has received considerable attention as the human body experiences repetitive shocks each time the foot hits the ground during running. These inputs generate vibrations to the musculoskeletal system. Although local or whole-body vibration is used as a training intervention as it may improve strength through neural adaptations [1], vibrations may have detrimental effects on the musculoskeletal structures [2, 3] and can also affect comfort [4] and performance [5]. Consequently, runners tend to minimise soft tissue vibrations (STV) by wearing adequate equipment (such as compressive apparel and footwear) and/or developing biomechanical [6] and neuromuscular adaptations [7, 8]. Reducing STV through these adaptations may also induce early neuromuscular fatigue [9]. No literature review has addressed STV during running yet, despite its evident importance from a comfort, performance and injury prevention perspective. Thus, the aims of this narrative review are to (1) provide a comprehensive overview of the literature on STV during running; (2) present several output parameters and explain the various approaches with their strengths and limitations for quantifying STV; (3) summarise the factors that affect STV.

Twenty-seven articles were obtained by searching the electronic bases of PubMed-NCBI, ScienceDirect, Web of Science and Google Scholar, using the following keywords in title/abstract linked with the following Boolean operators: Soft Tissue Vibrations AND Running AND (Fatigue OR Shoe OR Footwear OR Compression OR Muscle Tuning). Electronic database searching was supplemented by examining the references of 39 relevant articles. A total of 66 articles have been integrated for the present narrative review.

## Origin and Characteristics of Soft Tissue Vibrations in Running

### Background

**Fig. 1:**
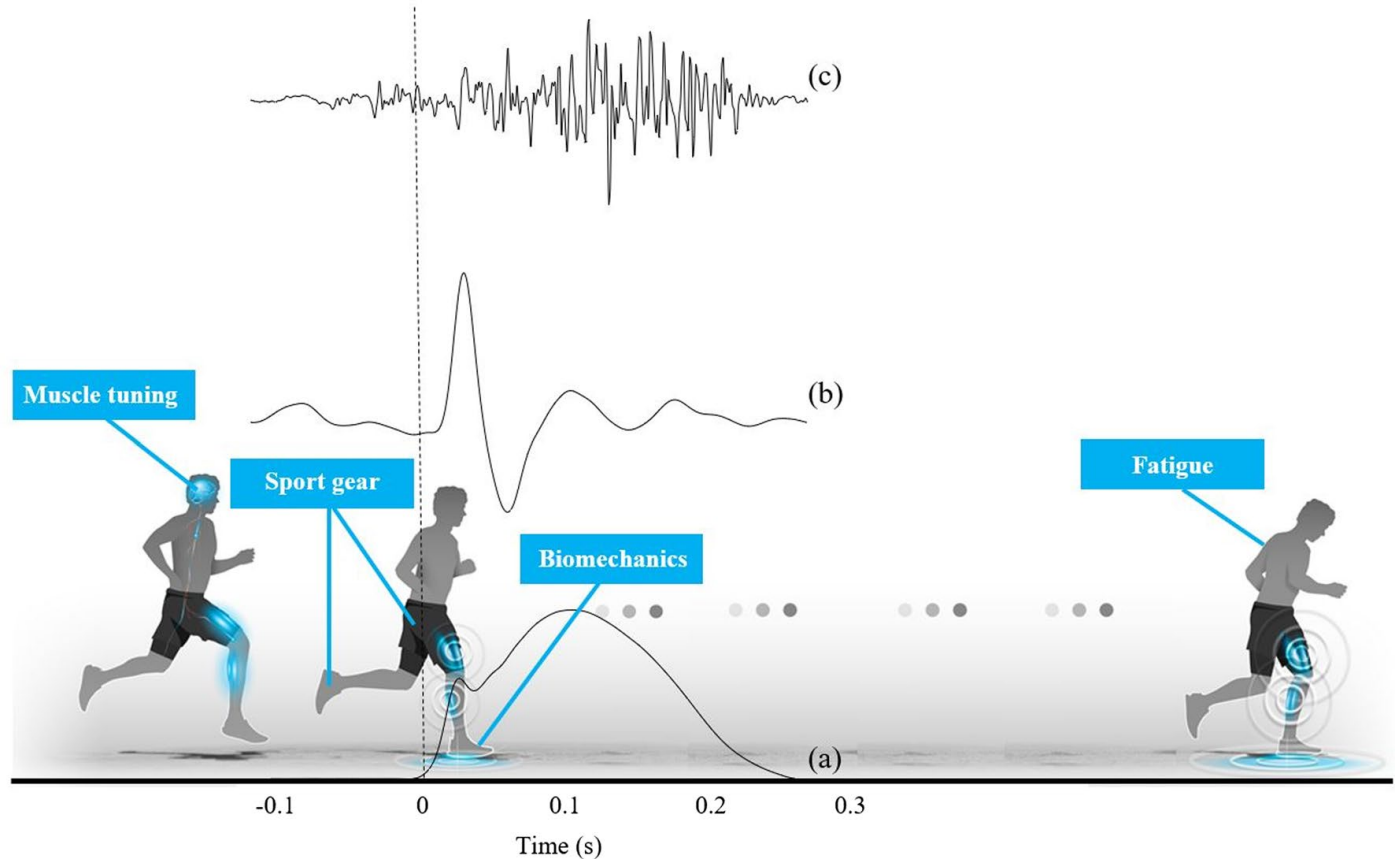
Factors affecting soft tissue vibrations: muscle tuning, foot strike pattern, sports gear and fatigue. Typical signals of vertical ground reaction force (**a**), *gastrocnemius medialis* (GM) acceleration (**b**) and electromyographic activity (**c**) are represented

**Fig. 2:**
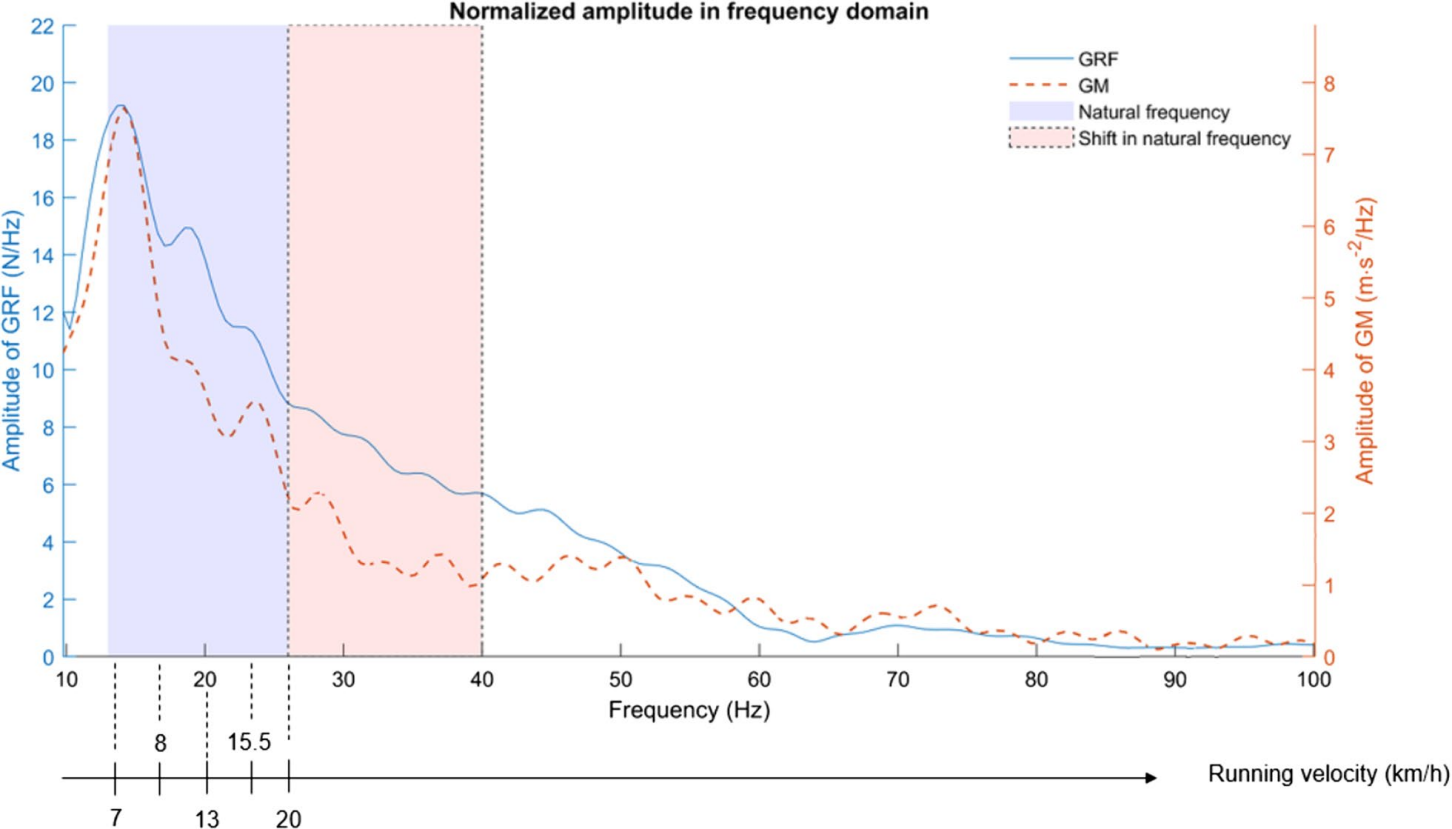
Frequency signals of ground reaction force (full line) and gastrocnemius acceleration (dotted line) during running. The blue area is equivalent to the natural muscle frequencies (Wakeling et al. [19]), which coincide with impact frequencies during different running velocities (from Trama et al. [26] and Boyer et al. [20]). Shifts in natural frequency are not sufficiently important to be outside the GRF frequency content

During running, each contact between the foot and the ground causes an impact, which is considered to be the initial input into the musculoskeletal system (Fig. 1a). The so-called ground reaction force is characterised by amplitude values between 1.5 and 3 times the runner’s body weight within 30 ms, resulting in an excitation frequency in the range of 10–30 Hz [10]. This involves a shock wave from the lower limb (Fig. 1b) to the head in bones and soft tissue, reaching 10 g [11–15]. Soft tissue (including muscles, tendons, adipose tissue, ligaments and skin) exhibits a composite structural organisation, resulting in a viscoelastic behaviour and thus, oscillates in all directions when exposed to a shock [16–18]. Soft tissue can be considered as oscillating masses connected by spring/dampers units, characterised by their frequency, amplitude and damping. The number of oscillations performed by soft tissue in one second corresponds to its frequency (in Hz). Each soft tissue package tends to vibrate at a specific frequency, called natural (or free) vibration frequency. The natural frequency of bones is significantly higher (200–900 Hz [19]) than those of soft tissue packages, that oscillate at about 15 Hz when relaxed [20]. A resonance phenomenon occurs when two signals have the same frequency ranges. As the frequency of impact forces during running coincide with the natural frequency of soft tissue [21], a resonance phenomenon occurs following the foot/ground impact and can amplify STV. The “muscle tuning” [8] could limit resonance through an increase in muscular activation before foot–ground contact to minimise STV [22, 23]. Two different mechanisms, damping or natural frequency shifts, have been reported. An increase in the damping coefficient (i.e. a more rapid damping of the vibration) has been more often reported than shifts in the natural frequency [20, 24] not substantial enough to move away from those due to the impact forces (Fig. 2). Finally, if muscles are not tuned properly by central nervous system [7] in response to input changes, STV amplitude increases [25]. Thus, countering this resonance phenomenon is a major issue for comfort, running performance, fatigue and injury prevention perspectives.

### Measurement Methods of Soft Tissue Vibrations

Vibration is generally defined as the variation of a physical quantity around an equilibrium. The current literature suggests different ways to quantify STV, either with skin-mounted accelerometers or passive filmed markers, the first method being the most widely used. It is worth mentioning that accelerometers and markers will provide two different physical quantities and therefore, two different aspects of the soft tissue motion. Indeed, a motion capture system will provide a displacement around a position while an accelerometer will quantify a variation in acceleration amplitude. Thus, we suggest clear indication whether magnitude refers to displacement or acceleration.

The accelerometer placement is important as acceleration of STV differs within each soft tissue compartment [26]. However, no standardised guidelines have been published yet. Video tracking with passive markers has also been used to quantify STV [27, 28]. However, only vibration displacement can be assessed with this method because of the low sampling frequency. Indeed, while most researchers have used a sampling frequency over 1000 Hz with tri-axial accelerometers, sampling frequency as low as 150 Hz has been widely used both with video tracking [28, 29] and accelerometers [27]. Even though the Shannon–Nyquist’s theorem is respected, the accuracy of these video and accelerometer tracking measurements remains questionable.

The choice between the two measurement methods (marker or accelerometer) should be based on the experimental design to be used. It has been shown that the two methods could be used to evaluate STV [30], by opting for markers only if the sampling frequency is greater than 500 Hz.

### Processing Methods of Soft Tissue Vibrations

In running, STV occurs in three dimensions: vertical—also called longitudinal, axial or proximo-distal; antero-posterior—also called transverse; and medio-lateral. Thus, one could speculate that soft tissue packages are 3 degree of freedom (DOF) systems. Some authors have analysed only the longitudinal axis [31, 32], which requires a careful alignment of the accelerometer along the longitudinal axis of the segment. Other studies highlighted the importance of analysing transversal components of the acceleration signal because vibration magnitude can be equally, if not more important along the transverse axis (e.g. peaks acceleration at approximately 9 g while running downhill [9]) compared to the longitudinal axis (10 g) [31, 32]. Such high transversal acceleration is explained by the anatomical structure of tissues weaker under shear than compression stress [16–18]. To the best of our knowledge, only few authors have quantified the resultant acceleration of soft tissue [9, 33]. It is worth mentioning that changes along one axis could be compensated by changes along other axes. For tibial vibrations measurement, it has been noticed that considering all axes, either individually or using the resultant (i.e. square root of the sum of squares of each axis) improves the repeatability and the reliability of the outcome variables (i.e. amplitude, frequency and damping) [34].

Along the three axes, amplitude, frequency, and damping of STV are reported in numerous studies since each of these three parameters brings different information.

- According to the Newton second law, the acceleration of the vibration provides information on the magnitude of stress undergone by the musculoskeletal system as:
  1 $$a = \frac{F}{m}$$
  with *a* the acceleration in ${\text{m}}\;{\text{s}}^{ - 2}$, *F* the force in ${\text{kg}}\;{\text{m}}\;{\text{s}}^{ - 2}$, and* m* the mass in kg.
  It is worth noting that both the word “*acceleration*” [14] and “*deceleration*” [6, 35] are used depending on the referential and/or direction studied.
  Using video tracking involves to deriving the position twice, which could amplify the error in the acceleration signal.
- The frequency corresponds to the number of oscillations undergone by soft tissue packages during one second (expressed in Hz), and as depicted by Eq. 2, gives information on the natural frequency of the soft tissue packages [36]:
  2 $$f = \sqrt{\frac{k}{m}} / \, 2\pi$$
  with *f* the frequency in Hz and *k* the stiffness in N m^−1^.
- The damping coefficient is rather an indicator of the magnitude of the adaptation after foot/ground impact (i.e. to what extent muscles tune their activity to minimise STV). By definition, damping represents the decay of the initial power over time and is expected to follow an exponential decay, $\exp^{ - c \times t} ,$ where c describes the damping coefficient and t the time [24]. A small damping coefficient indicates less damping needed [24]. Various terminologies are used in the literature, inducing a lack of understanding. For instance, a smaller damping coefficient could be associated with both of the following terms: a weaker one and a better one [14]. Conversely, a higher damping coefficient could correspond to a strong and worst damping [6]. In experimental studies, the damping coefficient is expressed in “per second”, with the notation s^−1^ being preferred to Hz, while in model studies, the damping coefficient of a structure is expressed in kg s^−1^ or N s m^−1^.

**Fig. 3:**
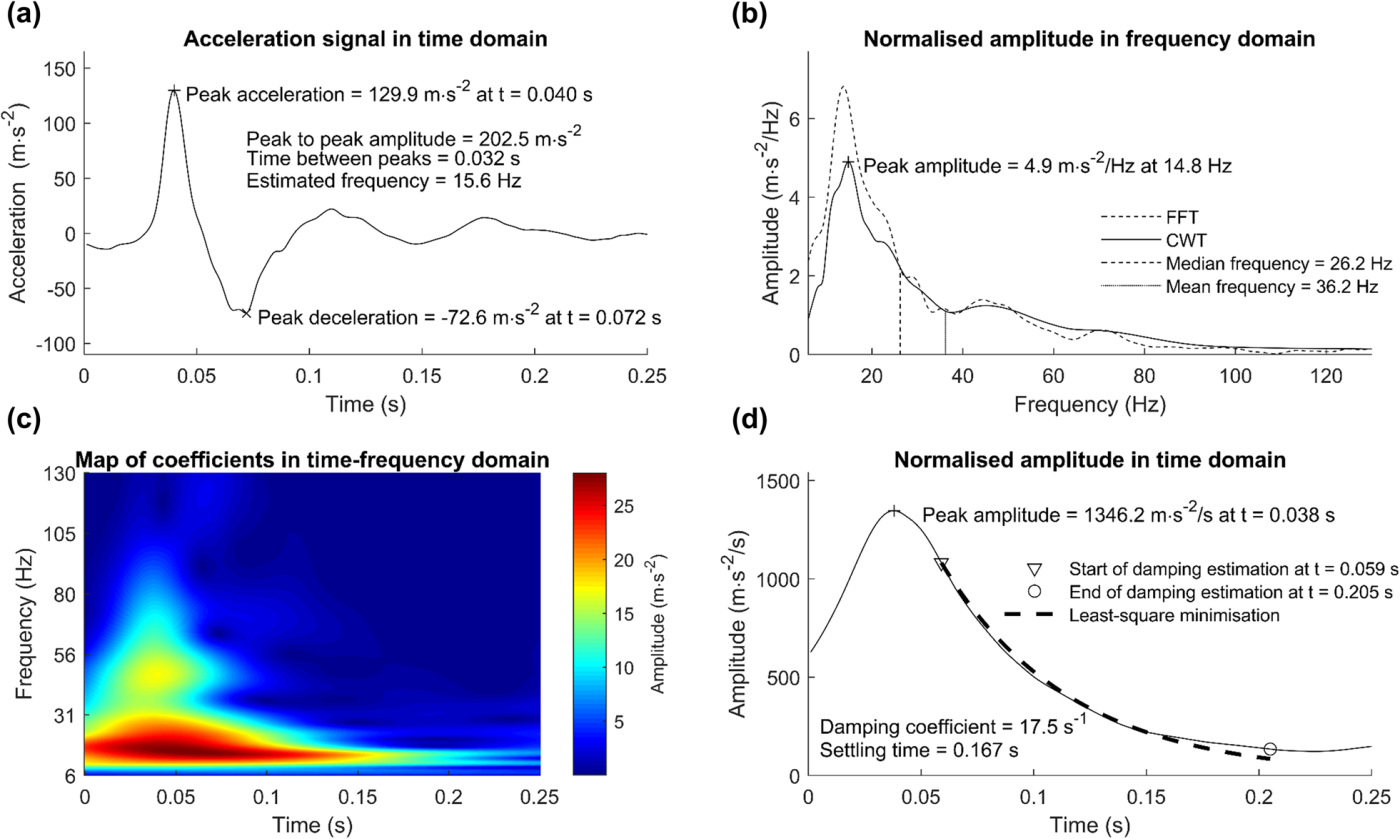
Authors’ data during running for the *gastrocnemius medialis* (GM): **a** amplitude variables obtained with time analysis; **b** frequency variables obtained by frequency analysis; **c** heat map (wavelet coefficients) resulting from time–frequency analysis; **d** damping variables in time domain resulting from time–frequency analysis

The vibratory signal of soft tissues during running is composed of several frequencies. The higher frequencies amplitude decreases further than the lower ones [15, 37]. Because of the multicomponent nature of the signal, several methods have been developed to identify the modal parameters of the signal and assess the three parameters above. Each of them can be described by different variables and obtained from different types of analysis (Table 1 and Fig. 3).

**Table 1:** Variables and type of analysis used in the literature to assess soft tissue vibrations

Type of analysis	Variable (unit)	Description/Computation	References
Temporal	Peak acceleration (m s^−2^)	Maximum of the absolute value of the acceleration signal	Trama et al. [32,62] Boyer and Nigg [22, 26, 34] Nikooyan and Zadpoor [61] Giandolini et al. [14]
	Root mean square acceleration (m s^−2^)	Average root mean square of the signal with a 0.01 s window	Ehrström et al. [9]
	Standard deviation of accelerations (m s^−2^)	Standard deviation of the acceleration signal	Gellearts et al. [27]
	Maximal displacement (m)	Difference between the maximum and minimum position of the soft tissue markers relative to the segment of interest	Borràs et al. [29] Broatch et al. [28]
Fast Fourier Transform	Normalised Fourier Transform (m s^−2^/Hz)	Fourier Transform normalised by the frequency resolution Represent the amplitude at each frequency	Boyer and Nigg [26, 34] Enders et al. [39] Khassetarash [36]
	Power Spectral Density (m^2 ^s^−4^/Hz)	Square of the Fourier Transform normalised by the inverse of the product of the signal length by the sample frequency Represent the power at each frequency	Giandolini et al. [14] Friesenbichler et al. [41]
Continuous Wavelet Transform	Time integral of wavelet coefficients for different frequency bands (m s^−2^/Hz)	Equivalent to the Normalised Fourier Transform	Trama et al. [15, 32]
	Sum of wavelets power	Non-indicated (normalised)	Khassetarash et al. [31]
Temporal	Peak frequency estimate	Inverse of the duration between the two first peaks after heel strike	Boyer and Nigg [22]
Fast Fourier Transform	Peak frequency (Hz)	Frequency with the most amplitude	Enders et al. [6] Giandolini et al. [14]
	Mean frequency (Hz)	Weighted average product of the amplitude of the frequency by the frequency	Friesenbichler et al. [41] Enders et al. [6]
	Median frequency (Hz)	Frequency that split the amplitude or power spectrum in half	Enders et al. [6] Trama et al. [62]
Continuous Wavelet Transform	Peak frequency (Hz)	See above	Trama et al. [15, 32, 62]
Continuous Wavelet Transform	Damping coefficient (s^−1^)	Decrement of logarithm power or logarithm decrement of power (estimation via least-square minimisation)	Enders et al. [6, 39] Khassetarash et al. [31, 36] Trama et al. [15, 32, 62]
	Settling time (s)	Time between maximum amplitude and 10% amplitude	Khassetarash et al. [31] Trama et al. [62]
	Energy dissipation (J kg^−1^)	Product of the time integral of the square vibration speed by the double of damping coefficient	
Partly Ensemble Empirical Mode Decomposition	Damping coefficient (s^−1^)	See above	Khassetarash et al. [40]
	Damping ratio (ø)	Damping coefficient relative to the critical damping of the frequency damped	
	Energy dissipation (J kg^−1^)	See above

#### Time Analysis

Time analysis (Fig. 3a) is the simplest method to analyse vibratory signals. From the time representation of the acceleration signal (Fig. 1b), one can compute the peak acceleration [14, 22, 32, 33, 38], the mean standard deviation [27], or the root mean square acceleration [9]. A temporal analysis of passive marker displacement allows the calculation of the maximum displacement of the soft tissue [28, 29]. With these amplitude characteristics, the intensity of the mechanical stress undergone by the soft tissue package can be quantified. The total vibration exposure was defined as the product between the vibration amplitude, duration, and stride frequency [31]. In addition, it is possible to estimate the frequency of the vibration, which corresponds to the inverse of the time period between two consecutive peaks [22]. Although this method is the simplest to implement, it is very sensitive to the filters applied on the raw signals. In running, one study has applied a low-pass filter at 50 Hz on the signal [30], while the others did not filter the signal. It is possible to compute damping from a temporal analysis (e.g. model or logarithm decrement between peaks) [20, 39]. However, other methods [40, 41] are preferred to compute frequency and damping of the vibration signal in running.

#### Frequency Analysis

The most common algorithm employed is the Fast Fourier Transforms (FFT). FFT allows the calculation of the Fourier Transform (FT), which is often normalised (nFT) by the frequency bin width (i.e. FFT resolution) to obtain physical values in the spectrum (Fig. 3b). By squaring the FT and dividing by the frequency resolution, it is possible to calculate the power spectral density of the signal (PSD). nFT represents the amplitude of the signal as a function of the frequency, in our case expressed in ${\text{m}}\;{\text{s}}^{ - 2} /{\text{Hz}}$, while PSD represents the power as a function of the frequency, in ${\text{m}}^{2} \;{\text{s}}^{ - 4} /{\text{Hz}}$ [14]. One of the main advantages is that PSD allows a comparison of the values from different studies that would not have used the same sampling frequency and/or frequency bin width. These spectra allow the calculation of the peak frequency (i.e. the frequency with the highest amplitude), peak amplitude or peak power [30, 37, 40, 42]. It is also possible to compute the median [6] or the mean [6, 42] frequency, which allows to characterise how the energy is distributed over the frequency continuum. If the peak, mean, and median frequencies are close to each other, the energy is localized near the peak frequency. However, if these metrics are far apart, the acceleration signal may be composed of several distinct frequencies. Knowing that muscle activity modulates STV frequency [8], it is of great interest to have a detailed description of the frequency characteristics of STV in order to presuppose the effect of a given condition (e.g. sports gear, footwear, fatigue) on muscle activation [22]. Subsequently, frequency analysis allows to calculate the total amplitude or power within a given frequency range by integrating the nFT or PSD curve, respectively [32, 43]. PSD and nFT are dependent on the vibration amplitude and duration; consequently, they could be more suitable to characterise the total vibration exposure compared to time analysis.

In addition, the transfer of amplitude between the input (i.e. impact quantified with ground reaction force or heel/tibial accelerometer) and the STV [15, 35], or between two locations on the same muscle [26] can be quantified. The transfer corresponds to the ratio between the two locations for one amplitude parameter, such as peak amplitude, PSD, or entire power spectrum. Transfer can be expressed as a percentage of variation [15] or as a transmissibility function expressed in dB [25]. Consequently, the transfer gives information about the relative movement between two regions of interest. Changes in transfer function, instead of changes in vibration amplitude, can better inform on the presence of neuromuscular adaptions induced by the muscle tuning [25]. Indeed, muscle tuning adaptations can lead to a decrease in the transfer of the vibration from the bones to soft tissue packages (i.e. the bones and soft tissue are more united).

#### Time–Frequency Analysis

The frequency content of the vibration signal changes over time as higher frequencies are more damped than lower ones [41], which has led authors to employ time–frequency analyses such as continuous wavelet transforms (CWT) [33, 37, 40, 42, 43] or empirical mode decomposition-based method (EMD) [41]. The difference between these two methods is that CWT works on all the frequency range, while EMD extracts intrinsic mode functions. The major limit of the CWT is the choice of the mother wavelet which can differ between studies. The limit of EMD is that mode functions are specific to the signal, meaning that their numbers and frequency are not identical for all signals. These limits complicate the comparisons between studies and/or individuals. Time–frequency analyses permit the quantification of the vibration amplitude evolution as a function of time and frequency (Fig. 3c). By choosing the wavelets or mode functions analysed, mainly between 6 and 130 Hz [32, 33, 37, 40] (the frequencies below and above corresponding to movement and noise, respectively), it is possible to calculate different indicators for different time and frequency ranges. To illustrate the major advantage of time–frequency analysis over the frequency analysis, one can consider a signal with a first frequency with high power during a short time period, and a second frequency with low power for a long time. The frequency analysis will depict a spectrum with two frequency peaks of similar energy, without the possibility to identify if the power of these frequencies were different. Concurrently, the time–frequency analysis will display a map of wavelet coefficients on which differences in power over frequency and time could be clearly identified. This information, though it is not yet widespread in literature, allows a better description of the vibration amplitude throughout the stance phase (i.e. braking, mid-stance, propulsion) and permits a better understanding of the effect of vibrations on the musculoskeletal and neuromuscular systems.

No study has used the map of wavelet coefficients (Fig. 3c) as a descriptive variable during running. Instead, maps are used to extract variables [32, 33, 37, 42]. First, by integrating the map in function of time, one can compute an equivalent of the nFT and obtain the same information as that obtained with FFT analysis. Although the frequency domain is well estimated by CWT (Fig. 3b), these methods are a compromise between the time and frequency resolution and thus, are often used in parallel of frequency analysis. Second, by integrating the map in function of the frequency [15] or by summing the wavelets power [6, 31, 33, 37, 40], one can compute the overall amplitude of the vibration in the time domain. The result of this computation depends on the frequency range analysed, and/or of the wavelets summed. As the theoretical overall power decay is known and linked to the damping properties of the soft tissues, it allows estimation of damping (Eq. 3). In addition, the same decay can be obtained from the envelope of the intrinsic mode functions computed with EMD [37].

3 $$p\left( t \right) = P_{0} e^{ - dt}$$

where $P_{0}$ is the power of the sum of the wavelets, *d* the damping coefficient, and *t* the time.

As the power decay is exponential, the logarithm of the decay is linear. The damping can be estimated with least-square minimisation, the damping coefficient being the slope of the regression line [6, 40, 41]. Another method is to use an optimisation function [15] to minimise the error between the measured decay and the modelled decay (Eq. 3 and Fig. 3d).

Other methods have been proposed to assess the damping of the vibration. For instance, one can calculate the settling time of the vibration that corresponds to the duration between the time at maximal power, and the time when the power is damped at 90% [31, 33]. The greater the damping, the shorter the settling time. The dissipation of energy, depending on the damping and speed, can be computed [31, 37, 41]. Several studies depicted that the higher frequencies of vibrations were more damped [15, 41] but dissipated less energy. Knowing that damping can be altered by foot strike pattern [6] or fatigue [31, 41], this indicator can inform on the neuromuscular adaptions and strategies to limit the vibration exposure.

To conclude this section, time–frequency analysis (i.e. continuous wavelet transforms or empirical mode decomposition) is the most common method for analysing vibration in running. However, the analysis of multicomponent signals with methods reducing the compromise of the time or frequency resolution has been employed in different areas. Use of these original approaches originating from synchro-squeezing transforms could be suitable for analysing running vibration data [44–46].

## Parameters Influencing Soft Tissue Vibrations

Both intrinsic (biomechanical and muscle patterns) and extrinsic (sports gear) parameters of the runner have an effect on STV (Fig. 1). Creation of functional groups will allow researchers to group individuals who react in a similar way to a specific sportwear intervention and see whether these runners have the same morphological characteristics.

### Foot Strike Running Pattern

Each runner automatically and naturally adopts a specific biomechanical pattern, which has been termed “preferred movement path” [6]. One macroscopic feature describing part of one’s preferred movement path is his/her foot strike pattern [47]. In level running, it is well known that runners strike the ground either with their heel (rearfoot strikers) or with their midfoot/forefoot. Foot strike patterns are characterised by differences in muscle activation, kinetics and kinematics [48–50]*.* Rearfoot strikers usually demonstrate a more extended knee and dorsiflexed ankle at foot strike. Conversely, greater knee flexors and plantar flexors activities, more flexed knee and more plantarflexed ankle at foot strike have been observed in midfoot/forefoot strikers [51]. Based on these considerations, all of these biomechanical differences could affect the shock wave propagation through the lower limb and all STV characteristics. Indeed, it has been observed that natural frequencies and damping of STV were affected by the knee flexion angle during isometric contractions [20]. In line with the preferred movement path paradigm, it is worth mentioning that STV damping on the *gastrocnemius medialis* (GM) was impaired when heel strikers were asked to run with a forefoot strike pattern and vice-versa [6]. It is suggested that runners demonstrate the best damping of STV with their preferred foot strike pattern.

### Running Speed

As running speed increases, the energy of impact increases and more energy needs to be dissipated in the soft tissue packages. Across a range of speed between 7.2 and 19.6 km/h, significant increases in STV amplitude (+ 144%), frequency (+ 27%) and damping (+ 27%) have been demonstrated [22, 32]. In addition, the increase in running speed is accompanied by greater ground impact magnitude and an increased level of muscle activity. This increase in muscle activity is accompanied by an increase in the stiffness of the soft tissue package, which influences the vibration frequency.

### Sports Gear

#### Compressive Garments

An effective method to attenuate STV during dynamic activities is the use of sports compressive garments. To the best of our knowledge, five studies investigated the effects of compressive garments on STV during downhill [9, 29] or flat running [27, 28, 52]. The main findings are summarised in Table 2. In the literature, two types of garments have been used when studying STV: tights compressing both calves and thighs, and shorts compressing thighs only. Vibrations have been measured with tri-axial accelerometers or 3D motion capture where the sensors (accelerometers or markers, respectively) were put either under [9] or on top of the garments [27–29, 52]. One could suggest that setting the device under the garments may affect the compressive pressure and/or applied an extra pressure on the device which could underestimate the benefit and/or distort the accelerations signals.

**Table 2:** Effect of equipment on soft tissue vibrations

	References	Participants details (Level|Sex|Age|Weight|Height)	COMP / TEST details (Type|Pressure (mm Hg)) / Shoe characteristics)	Running protocol	Device measuring STV|Locations|Frequency	Effects of COMP / TEST
*Apparel*						
Flat running	Coza et al. [51]	Young and active subjects M = 2|F = 2| n.i	n. i	Treadmill 5 speeds × 3 shoes per COMP	Tri-axial accelerometers VM, GL, BF 2400 Hz	Damping ↗ (+ 8%)
	Gellaerts et al. [27]	Healthy men | M = 11|25.1 ± 2.7 years|67.5 ± 1.3 kg|175.5 ± 1.8 cm	Shorts| COMP1 (Thigh): 20.4 ± 1.7 COMP2 (Thigh): 14.5 ± 1.3 COMP3 (Thigh): 9.6 ± 0.8	Treadmill 10 km/h 1 min per COMP	Tri-axial accelerometers on textile VM and RF 150 Hz	SD Ar ↘ (from − 5.15 to − 16.2%) for all muscles and garments
	Broatch et al. [28]	Recreational athletes| M = 14|27 ± 5 years|77.8 ± 8.4 kg|180.9 ± 7.3 cm	Tight | COMP1 (Thigh) 12.1 ± 2.3 (Calf) 17.2 ± 6.2 COMP2 (Thigh) 12.9 ± 2.7 (Calf) 14.6 ± 4.9 COMP3 (Thigh) 13.2 ± 3.1 (Calf) 11.4 ± 4.7	4 × 9 min continuous treadmill 3 min at 8 / 10 / 12 km/h) with 30 min rest	3D motion capture, Markers on textile VAS and GAS 250 Hz	v. MD ↘ (− 10%) for VAS for all garments m. l. MD ↘ (− 20%) for VAS for all garments v. MD ↘ (− 4%) for GAS for COMP1 ( m. l. MD ↘ (− 11%) for GAS for COMP1
Downhill running	Ehrstrom et al. [9]	Well-trained runners M = 13|38.6 ± 5.7 years|72.1 ± 4.7 kg|175.8 ± 5.1 cm	Tight | COMP (Thigh) 16–18 (Calf) 20–25	Outside 40 min DHR − 8.5% slope 55% VO2max	Tri-axial accelerometers under textile VL and GM 1000 Hz	RMS Ar ↘ (− 5%) only for VAS
	Borras et al. [29]	Moderately train soccer player M = 9||27.7 ± 10.9 years|76.1 ± 6.1 kg|176.8 ± 3.6 cm	Shorts| n. i	Treadmill 40 min DHR − 10% slope 85% VO2max	3D motion capture Markers on textile RF and VAS 200 Hz	MD ↘ (− 7.8%)
*Footwear*						
Midsole composition	Giandolini et al. [14]	Recreational and competitive runners|M = 12|42 ± 10 years|75.2 ± 8.4 kg|178 ± 4 cm	COND: Elastic (full EVA 55C-Asker) TEST: Viscous (bi-material 42 and 52C-Asker)	20-m indoor track|3.33 m/s|5 trials per shoe	Tri-axial accelerometers on VM and GM 2000 Hz	For GM: v. a_peak_ ↘ (− 8.2 ± 3.5%) v. PSD ↘ (− 17.5 ± 6.2%) v. f_peak_ ↔ v. Damping ↔ For VM: v. a_peak_ ↘ (− 12 ± 3.7%) v. PSD ↘ (− 19.7 ± 6.2%) v. f_peak_ ↔ v. Damping ↘ (− 19.3 ± 17.1%)
	Giandolini et al. [52]	n. i.|M = 8|n. i.|n. i.|n. i	COND: EVA, 60C-Asker (hard) TEST: eTPU 40C-Asker (elastic and soft)	treadmill|3.33 m/s|2 trials per shoe. during 5 min	Tri-axial accelerometer TA, GL, RF and BF 2560 Hz	For TA: v. PSD ↗ (+ 26.7 ± 20%) For RF: a.p. PSD ↗ (+ 42.6 ± 35.5%) For GL and BF: PSD ↔
	Boyer et al. [22]	Regularly runners|M = 10|25 ± 4.2 years	COND: Elastic (52C-Asker) TEST1: Viscous (50C-Asker) TEST2: Visco-elastic (40C-Asker) TEST3: Visco-elastic (55C-Asker) TEST4: Visco-elastic (70C-Asker)	16-m indoor track| 2, 3, 4 and 5.5 m/s| 3 trials per shoe and surface	Tri-axial accelerometers TA, GM, VL and BF 2400 Hz	For all muscles: v. a_peak_ ↔ v. f_peak_ ↔
Midsole shape	Trama et al. [32]	Recreational runners|M = 20|23.9 ± 2.1 years|73.6 ± 7.4 kg|177 ± 5 cm	COND: Flat ($h_{{{\text{toe}}}}$ = 40 mm, $h_{{{\text{heel}}}}$ = 55 mm, ∆h = 15 mm) TEST: Rocker ($h_{{{\text{toe}}}}$ = 41 mm, $h_{{{\text{heel}}}}$ = 58 mm, ∆h = 17 mm)	Indoor track| 8, 10.5, 13 and 15.5 km/h|5 trials per shoe and running speed	Tri-axial accelerometers GM and VL 1000 Hz	For VL and GM: v. a_peak_ ↔ v. f_peak_ ↔ Damping ↔
	eaGellaerts et al. [27]	Healthy men|M = 11|25.1 ± 2.7 years|67.5 ± 1.3 kg|175.5 ± 1.8 cm	COND: minimalist ($h_{{{\text{toe}}}}$ = 4 mm, $h_{{{\text{heel}}}}$ = 4 mm, ∆h = 0 mm) TEST: maximalist ($h_{toe}$ = 29 mm, $h_{{{\text{heel}}}}$ = 33 mm, ∆h = 4 mm)	Treadmill| 10 km/h| 1 min per shoe	Tri-axial accelerometers RF and VM 150 Hz	For RF: MD ↘ (− 20.6 ± 6.6%) For VM: MD ↘ (− 18.8 ± 2.5%)

List of Abbreviations present in the table: *M* Male, *F* Female, *n. i*. non-indicated, *COMP* Compression Tested, *COND* Condition, *TEST* Shoe Tested, $h_{{{\text{toe}}}}$: Height under the toe, $h_{{{\text{heel}}}}$: Height under the heel, ∆h: Height difference between heel and toe, *DHR* Downhill running, *VM* *Vastus Medialis*, *VL* Vastus Lateralis, *GM* Gastrocnemius Medialis, *GL* Gastrocnemius Lateralis, *BF* Biceps Femoris, *RF* Rectus Femoris, *VAS* Vastii, *GAS* gastrocnemii, *TA* Tibialis Anterior, *SD Ar* Standard Deviation of Acceleration resultant, *RMS Ar* Root Mean Square of Acceleration resultant, *MD* Muscle Displacement, a_peak:_ peak acceleration, f_peak:_ peak frequency, PSD: Power Spectral Density, v.: vertical, a. p.: anterior–posterior, m. l.: medio-lateral, ↘: significant decrease, ↔ : no significant change, ↗ significant increase

All studies, both during flat and downhill running, noticed up to a 20% decrease in STV when the pressure exerted by the garments increased. The observed differences in STV between garments seem to be independent of the amount of pressure [28]. For instance, the coefficient of determination for the relationship between the degree of pressure and the reduction in oscillation at 10 km/h for the vasti muscles was estimated to be 0.4 [27, 28]. As pressure was measured at rest and not during running, it could be suggested that this coefficient remains relatively low and there is a state of uncertainty on the garment’s capacity to maintain pressure during running [28]. The authors suggested that further research should investigate the amount of pressure by garments during running.

Compressive garments have been shown to be more effective in reducing oscillations on vasti muscles than gastrocnemii muscles [28]. Soft tissue masses are greater for thighs than for calves. Given the elastic nature of muscle and surrounding soft tissues, the capacity for soft tissue displacement following foot strike is likely greater for bigger soft tissue masses. Therefore, compression is likely to have a greater damping effect on vasti muscles.

It has been shown that compressive garments also minimise the harmful effects of impact forces during running. Indeed, for a given downhill running bout (slope: –8.5°, 40-min at a velocity associated with 55% of VO_2max_ [9]), perceived muscle soreness measured one day after the running test was found to be significantly lower for the quadriceps. Additionally, for a similar downhill running protocol (slope: − 10°, 40-min at a velocity associated with 85% of VO_2max_ [29]), muscular biopsies performed 48 h after running test highlighted higher inflammation and sarcomere injuries without compression. Moreover, the decrease in STV also induced a faster recovery of peripheral and central neuromuscular function and a lower impairment in running economy [9]. Overall, compression apparels seem to minimise STV and muscle activity in running leading to a reduction in soft tissue damage and fatigue. Whether muscle tuning and soreness are related remains unknown. As wearing compression garment decreases muscle activation and STV amplitude [28], one could speculate that the requirement for ‘muscle tuning’ is lower and may reduce neuromuscular fatigue.

#### Footwear

Footwear characteristics have the potential to influence impact and STV. Five studies have assessed the effects of changing (a) midsole materials (viscoelasticity and/or hardness) and (b) midsole geometry (thickness and/or curvature) on STV during running (Table 2).

#### Midsole Materials

*Viscoelasticity:* A viscous midsole has been found to reduce initial vibratory input of STV for calf and thigh muscles [14, 53]. In the viscous condition, the vertical amplitude decreased by 8–12% for the *vastus medialis* (VM) and *gastrocnemius medialis* (GM), equivalent to an about 1 g decrease in the acceleration peaks [14]. In addition, the vertical PSD was reduced by ~ 20–27% for GM, VM and the *tibialis anterior* (TA). Concerning viscoelasticity effect on the damping mechanism, there was a better damping for the viscous midsole condition for VM. However, it seems that changing midsole viscoelasticity has no significant effect on vibration frequency. It is worth mentioning that changes in leg STV amplitude and damping were associated with a decrease in EMG activity. Indeed, EMG activity before foot strike was lower for the viscous condition which might explain the better damping and the reduction in the amplitude of STV [14].

*Hardness*: It has been shown that changing midsoles hardness from Asker 40C (soft) to 70C (hard) did not change STV amplitude and frequency [22]. Even if harder shoes elicit higher impact peak force and loading rate, no significant effects of hardness were found on peak acceleration and peak frequency. These results agree with a model studying the effects of footwear on impact forces and vibration during running. It has been proposed that the central nervous system guarantees an acceptable vibration threshold above which central functions (e.g. vision, vestibular function) may be impaired through muscle tuning [13, 54, 55]. It has been shown that damping vibration remained similar between soft (Asker-56C) and hard (Asker-64C) shoes across all participants with a wide range of anthropometrics [24]. This is inconsistent with a previous model suggested that soft shoes showed a 2 to 3 times greater damping coefficient than hard shoes [7]. However, this study did not clarify hardness values and used greater speeds.

#### Midsole Geometry

No significant effect of midsole curvature (rocker) was found on STV, ground reaction forces or EMG activity along the longitudinal axis [32]. When comparing minimalist to maximalist footwear along the three axes, the latter being characterised by a thick and curved midsole, muscular oscillations were about 19% lower with maximalist shoes compared to minimalist ones [27]. Thus, large changes in midsole geometry affect STV. However, in this study, both midsole thickness and curvature were different so that no definitive conclusion can be made on the independent effect of midsole curvature and thickness on STV.

In summary, vibrations responses to footwear changes are more subtle and less obvious than for garments. To better understand what is happening at the soft tissue level, a combination of EMG and STV analyses might be beneficial.

#### Functional Groups

As the same shoe modification produces different reactions among runners (i.e. subject-specific results), the concept of functional groups has been proposed. In footwear science, a functional group is defined as a group of subjects who respond similarly to a specific shoe intervention [56]. A recent study recorded STV of the GM during 5-min treadmill run [57]. When running with the same shoe condition, the 42 participants demonstrated very different degrees of damping, without changes in muscle activation, input or natural frequency between the groups. These same researchers went further by studying, only along vertical axis, damping response to footwear changes on 32 runners presenting a wide range of anthropometrics [24], but no significant differences were found. However, their results of anthropometrics on vibration damping were insignificant, but runners with better damping in hard shoe showed a trend of a slightly greater body mass index. Thus, we suggest in addition either kinematics (i.e. foot strike pattern [6, 47]) or subject-specific anthropometrics (i.e. skinfolds, soft tissue masses, natural frequencies [29, 58]) assessments to elucidate their potential contribution to vibrations. Indeed, not all individuals have the same anthropometrics and muscle characteristics. Powerlifters had a higher STV frequency than runners [59], which was explained by a greater muscle stiffness or less subcutaneous fat layer [60]. Moreover, the population has been divided into participants who primarily damp and others who shift their natural frequency, which increases the likelihood that neuromuscular responses to interventions differ between these two groups [59].

When testing sports gear, we suggest defining functional groups based on both anthropometric measurements and STV characteristics measured in a controlled condition. Functional groups can be determined by using methods such as principal component analysis [58] or vector representation [56], as these are powerful and promising tools to identify clusters of subjects with similar characteristics [61].

## Effects of Fatigue on Soft Tissue Vibrations

Muscle tuning has been shown to occur in non-fatigued muscle [20, 22, 23]. However, muscle tuning requires an increase in muscle activity that could be impaired by fatigue. Whether or not muscle tuning is affected by neuromuscular fatigue is a relevant question. Six studies published between 2011 and 2022 focused on the effects of fatigue on STV [9, 31, 33, 41, 42, 62] (Table 3). The protocols employed to induce fatigue can be classified in four categories:

- A computer model with implemented fatigue [62],
- Level running until fatigue [31, 41, 42],
- Mountain trail running races of various distances (from 40 to 171 km) [33],
- Downhill running [9].

**Table 3:** Effect of fatigue on soft tissue vibrations

Reference	Participants details (Level|Sex|Age|Weight|Height)	Running protocol (Surface|Intensity|Duration|End of protocol)	Device measuring STV| Locations|Frequency|Time measurement	Effects of Fatigue
*Downhill*				
Ehrström et al. [9]	Well-trained male runners|M = 13|38.6 ± 5.7 years|72.1 ± 4.7 kg|175.8 ± 5.1 cm	Treadmill with − 8.5° slope **|** 55% ${\text{VO}}2_{\max }$ = 4.2 ± 0.3 m/s**|** 40 min|40 min	Tri-axial accelerometers VL and GM 1000 Hz Non-FAT: 4–5 min and 9–10 min FAT: 34–35 min and 39–40 min	For VL: RMS Ar ↗ (11.6 ± 5.9%) For GM: RMS Ar ↔
*Flat exhaustion*				
Friesenbichler et al. [41]	Recreational runners | M = 3|26.7 ± 2.3 years|65.3 ± 3.3 kg|173.8. ± 3.8 cm F = 7|31.7 ± 7.3 years|60.1 ± 6.4 kg|165.5 ± 4.3 cm	230-m outdoor track**|**F: 3.1 ± 0.2 m/s, M: 3.8 ± 0.3 m/s|10.4 ± 2.4 km **|** No longer able to maintain the required speed for 3 consecutive laps (690 m)	Tri-axial accelerometer TS 2400 Hz Non-FAT: 50 steps during the first 5 laps FAT: 50 steps during the last 5 laps	For TS: v. PSD ↗ (n.i) v. $f_{{{\text{mean}}}}$ ↔ Time to peak ↗ (n.i.)
Khassetarash et al. [40]	Professional male runners | M = 8|26 ± 3.6 years|65 ± 12 kg|175 ± 6 cm	Treadmill|4 m/s|9.6 ± 1.2 km **|** No longer able to run at the given speed or running distance more than 10 km	Tri-axial accelerometer GM 2000 Hz Non-FAT: first minute FAT: last minute	For GM: v. $f_{{{\text{amplitude}}}}$ ↗ (between 20 and 300%) Damping ↗ (20.3 ± 2.3%)
Khassetarash et al. [31]	Semi-professional middle- and long-distance male runners | M = 11|32.7 ± 9.94 years|65.9 ± 9.86 kg|174.3 ± 4.63 cm	Treadmill|4.3 ± 0.3 m/s|3.873 ± 1.147 km**|** No longer able to maintain their preferred high level of effort speed	Tri-axial accelerometer GL 2000 Hz Non-FAT and FAT: 5 strides average within 10 equal intervals (88 ± 26 s)	For GL $v \cdot a_{{{\text{peak}}}}$ ↗ (29.8 ± 3.44%) Damping ↗ (15.5 ± 0.65%) Energy dissipation ↗ (70.6 ± 0.49%) Settling time ↔
*Mountain trail running*				
Trama et al. [62]	Fifty-two experienced ultra-marathon runners M = 32|36.0 ± 8.1 years|70.3 ± 9.1 kg|178.3. ± 6.5 cm F = 20|36.9 ± 8.4 years|56.5 ± 6.1 kg|165.7 ± 6.7 cm	Treadmill|2.8 m/s|30 s	Tri-axial accelerometers VL and GM 2000 Hz Non-FAT: Pre-race (1 to 3 days before) FAT: Post-race (as soon as possible after the race)	For VL: $a_{{{\text{peak}}}}$_↘_ Damping _↘_ $f_{{{\text{peak}}}}$_↘_ Settling time ↔ For GM: $a_{{{\text{peak}}}}$ ↔ Damping ↔ $f_{{{\text{peak}}}}$ ↔ Settling time ↔

List of Abbreviations present in the table: *M* Male, *F* Female, *n. i*. non-indicated, *VL* Vastus Lateralis, *GM* Gastrocnemius Medialis, *GL* Gastrocnemius Lateralis, *TS* Triceps Surae, *Non-FAT* non-fatigued state, *FAT* fatigued state, *RMS Ar* Root Mean Square Acceleration resultant, *PSD* Power Spectral Density, $f_{{{\text{mean}}}}$: Mean frequency, $f_{{{\text{amplitude}}}}$_:_ Amplitude frequency, $a_{{{\text{peak}}}}$_:_ Peak acceleration, v.: vertical, ↘: significant decrease, ↔ : no significant change, ↗ significant increase.

The predictions of the computer model confirmed the hypothesis that the protective mechanism of muscle tuning is altered with fatigue. This predicted that fatigue will induce an increase in vibration amplitude and a worst damping, but no change in peak impact. It has been proposed that neuromuscular fatigue induced by short and intense running, impacting more specifically the fatigable type II fibres, can decrease muscle function and its damping capacity. In experimental studies [31, 41], an 15–20% increase in damping was noted.

The amplitude of vibrations, which is the only parameter that was systematically studied across the six studies, increased by 10–30% [9, 31, 41, 42], as expected by the model [62]. The only exceptions were for the GM after downhill protocol and mountain trail running races where amplitude was not modified [9, 33]. To explain these latter results, it was suggested that downhill and mountain trail running were more strenuous for knee extensor muscles as a consequence of greater braking forces, and therefore, altered more the *vastus lateralis* (VL) than the GM, which may not be the case in level running. The causes of the increase in vibrations amplitude are not yet fully understood. Contrary to the model prediction [61], concomitant increases in the passive peak of ground reaction force and vibration amplitude have been reported [31]. Moreover, if the increase in vibrations amplitude was solely due to change in impact forces, all muscles should behave similarly, which was not automatically the case [9]^.^ After efforts inducing neuromuscular fatigue similar to downhill running, impact intensity was not modified, either when quantified with force plates [63, 64] or tibial accelerations [65]. Since a greater vibration amplitude was denoted without kinematics alterations after short and intense exercises, these results confirmed that the protective mechanism of muscle tuning is altered by fatigue.

Among the five experimental studies on fatigue and STV, only two of them [9, 33] quantified the peripheral and central origins of neuromuscular fatigue. In the other experiments on short and intense running exercise [31, 41, 42], neuromuscular fatigue was not assessed and runners were considered fatigued when unable to maintain the pace. One experimental study quantified changes in vibration frequency with fatigue and found no changes in the vibration peak frequency [42]. As the vibration frequency is related to the mass and stiffness of the muscle (Eq. 2) [36], the stability of vibration frequency in the fatigue state may reflect that the soft tissue stiffness and mass were not altered in fatigued state. However, no definitive conclusion can be made as only two studies investigated this parameter, and more studies are needed to confirm frequency stagnation with fatigue [66].

## Conclusion

This review presents evidence that, from a methodological point of view in running biomechanics, skin-mounted accelerometers at a sampling frequency equal or greater than 1000 Hz are reliable sensors to quantify STV. In addition, all acceleration axes, and/or the resultant acceleration, should be analysed to extract, at least, one parameter for the amplitude, frequency and damping. To this end, recent methods have been developed to analyse the signal with a time–frequency approach. An important outcome of this review is that muscular (i.e. muscle tuning) and biomechanical (i.e. preferred movement path) adjustments minimise STV. In addition, compression apparels seem to be effective in reducing STV while responses to footwear interventions are highly subject-specific. Thus, shoe companies must better adapt the characteristics of the shoes to runners’ anthropometrics through, for instance, a complete biomechanical assessment to better understand how running kinematics may affect STV properties. Fatigue was found to increase vibration amplitude but should be investigated during prolonged running exercises by including an evaluation of neuromuscular fatigue and its etiology (central vs peripheral components). These future research priorities could prevent discomfort, muscle damage and running related injuries. It is important to note that this review is a narrative, not a systematic review, and hence, reflects the opinions and subjective assessment of the authors.

## Data Availability

Not applicable.

## Abbreviations

CWT: Continuous wavelet transform
EMD: Empirical mode decomposition
EMG: Electromyography
FT: Fourier transform
FFT: Fast Fourier transform
GM: *Gastrocnemius Medialis*
nFT: Normalised Fourier transform
PSD: Power Spectral Density
VM: *Vastus Medialis*
STV: Soft tissue vibrations
